# Divergent Effects of Systemic and Intracollicular CB Receptor Activation Against Forebrain and Hindbrain-Evoked Seizures in Rats

**DOI:** 10.3389/fnbeh.2020.595315

**Published:** 2020-11-17

**Authors:** Victor R. Santos, Robert Hammack, Evan Wicker, Prosper N’Gouemo, Patrick A. Forcelli

**Affiliations:** ^1^Department of Pharmacology and Physiology, Georgetown University School of Medicine, Washington, DC, United States; ^2^Department of Morphology, Federal University of Minas Gerais, Belo Horizonte, Brazil; ^3^Department of Cellular and Integrated Physiology, University of Texas Health Science Center at San Antonio, San Antonio, TX, United States; ^4^Department of Pediatrics, Georgetown University School of Medicine, Washington, DC, United States; ^5^Interdisciplinary Program in Neuroscience, Georgetown University, Washington, DC, United States; ^6^Department of Physiology and Biophysics, Howard University College of Medicine, Washington, DC, United States; ^7^Department of Neuroscience, Georgetown University School of Medicine, Washington, DC, United States

**Keywords:** cannabinoid, superior colliculus (SC), area tempestas, piriform cortex, genetically epilepsy prone rat (GEPR)

## Abstract

Cannabinoid (CB) receptor agonists are of growing interest as targets for anti-seizure therapies. Here we examined the effect of systemic administration of the CB receptor agonist WIN 55,212-2 (WIN) against audiogenic seizures (AGSs) in the Genetically Epilepsy Prone Rat (GEPR)-3 strain, and against seizures evoked focally from the *Area Tempestas* (AT). We compared these results to the effect of focal administration of the CB1/2 receptor agonist CP 55940 into the deep layers of the superior colliculus (DLSC), a brain site expressing CB1 receptors. While systemic administration of WIN dose-dependently decreased AGS in GEPR-3s, it was without effect in the AT model. By contrast, intra-DLSC infusion of CP 55940 decreased seizures in both models. To determine if the effects of systemic WIN were dependent upon activation of CB1 receptors in the DSLC, we next microinjected the CB1 receptor antagonist SR141716, before WIN systemic treatment, and tested animals for AGS susceptibility. The pretreatment of the DLSC with SR141716 was without effect on its own and did not alter the anti-convulsant action of WIN systemic administration. Thus, while CB receptors in the DLSC are a potential site of anticonvulsant action, they are not necessary for the effects of systemically administered CB agonists.

## Introduction

The epilepsies, as a group, are one of the most common neurological disorders and are associated with substantial morbidity, mortality, and economic burden. Identifying therapies to treat epilepsies can be a challenge due to the diversity of the condition. Epileptic seizures can arise in a variety of brain networks, resulting in differing semiology, pharmacosensitivity, and degree of impairment—a drug that works for one type of seizure may be ineffective or exacerbate another. Studies suggested that the deep layers of the superior colliculus (DLSC) are part of the network that generates audiogenic seizures (AGSs), a form of reflex seizures triggered by intense sounds (Faingold and Randall, 1999b). While AGSs are uncommon in humans, they can serve as a model for brainstem seizure activity. In rats and mice, bilateral lesions of the superior colliculus, microinjection of GABA antagonist (Depaulis et al., 1990), *N*-methyl-D-aspartate (NMDA) antagonist (Faingold and Casebeer, 1999a; Raisinghani and Faingold, 2003) or alpha1-noradrenergic agonist (Faingold and Casebeer, 1999a) markedly attenuated AGS severity (Merrill et al., 2003). In a different approach, optogenetic activation of DLSC also attenuates seizures in Genetically Epilepsy-Prone Rat (GEPR) 3 substrain (Soper et al., 2016; Wicker et al., 2019). Activation of the DLSC also potently suppresses seizures in other models of epilepsy, including the maximal electroshock model (Redgrave et al., 1992; Gale et al., 1993), evoked and genetic absence seizure models (Nail-Boucherie et al., 2002, 2005; Soper et al., 2016); the *Area Tempestas* (AT) model of forebrain seizures (Soper et al., 2016), and the pentylenetetrazole model of generalized seizures (Weng and Rosenberg, 1992; Soper et al., 2016). Further elucidating the neurotransmitter systems in the networks for brainstem and forebrain seizures may offer novel therapeutic targets for the treatment of generalized seizures.

The endocannabinoid system has shown promise as a target for seizure control, but there are conflicting reports about the efficacy of cannabinoid (CB) receptor targeting compounds in suppressing seizures (Rosenberg et al., 2017). One of the most promising candidates are CB1 receptor agonists, which exert anticonvulsant effects against generalized tonic seizures in the maximal electroshock test (Luszczki et al., 2006; Florek-Luszczki et al., 2014b; Tutka et al., 2018), but produce mixed effects against generalized seizures evoked by pentylenetetrazole (Bahremand et al., 2008a; Andres-Mach et al., 2012; Vilela et al., 2013; Aghaei et al., 2015; Huizenga et al., 2017) and limbic seizures in the kainic acid model (Bojnik et al., 2012; Shubina et al., 2015). These models are characterized by different types of seizures: the former by tonic seizures that critically rely on brainstem networks (Merrill et al., 2003, 2005), and the later by limbic seizures that critically rely on forebrain networks (Browning et al., 1993).

The CB1 receptor is the major CB receptor found in the central nervous system, and is coupled to Gi signaling cascades; its activation is typically associated with decreased transmitter release. Endocannabinoid signaling mediated through presynaptic CB1 receptors reduces both glutamate and GABA release (Kathmann et al., 1999; Sullivan, 1999; Hájos et al., 2000), and therefore is a potent regulator of neuronal excitability. Somewhat paradoxically, in the context of seizure suppression, the CB1 receptor co-localizes with GABA neurons in many brain regions it has been suggested that the primary cell type that expresses CB1 receptor is inhibitory (Katona et al., 1999, 2001). This is of particular interest in the context of the DLSC, as *disinhibition* of the DLSC by silencing GABAergic inputs from the substantia nigra *pars reticulata* (SNpr) is also potently anticonvulsant (Wicker et al., 2019). Both the DLSC and the SNpr express CB1 receptors, with the SNpr displaying some of the highest receptor density in the brain (Herkenham et al., 1991). Thus, here, we sought to determine the effect of CB1 receptor agonists on brainstem seizures in GEPR-3 rats and forebrain seizures in the Area Tempestas model while comparing the effects of systemic and intra-DLSC delivery of CB1 agonist.

## Materials and Methods

### Animals

Experiments were performed on male Sprague–Dawley (SD) purchased from Envigo (Frederick, MD, USA) rats and GEPR-3s obtained from a colony maintained at Georgetown University. Animals weighed 270–285 g at the time of surgery and were housed two per cage in the Georgetown University Division of Comparative Medicine under environmentally controlled conditions (12 h light/dark cycle, lights on between 6:00 AM and 6:00 PM; ambient temperature 23°C ± 1°C; controlled humidity) with food (Lab Diet, #5001) and water provided *ad libitum*. All the procedures and experiments were conducted following the Association for Assessment and Accreditation of Laboratory Animal Care standards, the Guide for the Care and Use of Laboratory Animals (Rowan, 1979), and were approved by the Georgetown University Care and Use Committee.

### Surgery

Surgeries were performed as previously described (Santos et al., 2018). Briefly, rats were anesthetized with equithesin (2.8 mg/kg, i.p.) and placed into a stereotaxic frame (David Kopf, Tujunga, CA, USA). Surgery was conducted with the incisor bar at +5.0 mm above the interaural line according to the atlas of Pellegrino and Cushman (1967). For all microinjection experiments, a 22 g guide cannula (Plastics One, Roanoke, VA, USA) was paired with a 28 g internal cannula with the internal cannula extended 2 mm deeper than the guide. A screw cap was placed on the guide cannula to keep cannula tracks clean. Cannulae were implanted unilaterally into the AT (Piredda and Gale, 1985; 4.0 mm anterior to Bregma, 3.5 mm lateral to the midline, 5.5 mm ventral to the dura) and/or into the DLSC (5.0 mm posterior to Bregma, 2.5 mm lateral to the midline, and 4.5 mm ventral to the dura; Pellegrino and Cushman, 1967). Atlas panels shown in the Figures are reproduced in modified form from the Brain Maps 4.0 atlas (Swanson, 2018). Cannulae were fixed in place with jeweler’s screws and dental acrylic. Following surgery, all animals were given at least 1 week to recover before behavioral testing.

### Systemic Drug Administration

The CB1 receptor agonist, WIN [WIN 55212-2 mesylate (R(+)-[2,3-dihydro-5-methyl-3-[(morpholinyl) methyl]pyrrolol[1,2, 3 de]-1,4-benzoxazinyl]-(1-naphthalenyl) methanone)], was obtained from Tocris Biosciences (Bristol, UK) and dissolved in 50% of DMSO and 0.9% saline. Solutions were prepared at a concentration of 2 mg/ml and drug or vehicle was administered by intraperitoneal (i.p.) injection at a volume of 1 ml/kg. The drug was administered 20 min before seizure testing.

### CB Drug Microinfusion

The CB1/2 receptor agonist CP 55940 (CP) was dissolved in 50% DMSO-50% saline, and 26.5 nmol (in 0.5 μl) was microinfused bilaterally into the DLSC. We selected CP 55940 over WIN for microinjection, because it has a higher affinity for the CB1 receptor than does WIN. By contrast, we selected WIN for systemic administration because its anticonvulsant profile has been better characterized than that of systemic CP. The CB1 receptor antagonist SR 141716A (SR, rimonabant) was dissolved in 50% DMSO-50% saline, and 1 μg (4.3 nmol) in 0.5 μl was infused into the DLSC. These drug doses were chosen based on published reports (Sañudo-Peña et al., 2000; Citraro et al., 2013; Florek-Luszczki et al., 2014a). Drug microinfusions were performed essentially as we have previously described (Wicker and Forcelli, 2016; Wicker et al., 2019). In brief, rats were gently restrained and a 28-gauge internal cannula was inserted. Drug infusion was controlled by a syringe pump driving a Hamilton syringe programmed to deliver the drug at a rate of 0.2 μl/min. After the completion of the infusion, the internal cannula was left in place for at least 1 min to reduce drug reflux up the cannula track and then removed. Drug microinfusion was performed 5 min before seizure testing.

### Piriform Cortex (Area Tempestas) Seizures

Twelve SD rats were used for these experiments. A stainless-steel guide cannula was stereotaxically implanted above the left AT as described above. Bicuculline methiodide (BMI; Sigma–Aldrich) was dissolved in 0.9% saline at a concentration of 1 mM, and a dose of 100–280 pmols (0.2 μl/min) was used to induce seizures. Bicuculline was injected immediately following intra-DLSC injections or 20 min following systemic WIN. Initially, a 100 pmol dose of bicuculine was microinjected into the AT; in the absence of seizure activity, the amount of bicuculline injected was increased (increments of 20 pmol) on a subsequent day until a seizure with the score of 3–5 on the modified Racine scale (see below) was reached. After AT injections were completed, SD rats were monitored for 60 min in a transparent plastic box, and behaviors were documented by an observer in real-time. In some SD rats, electroencephalogram (EEG) was used to monitor electrical activity correlated with behavioral seizures. During stereotaxic surgery, six holes were made in the skull so that six EEG screw wire electrodes could be screwed in for dura-mater contact. Placement of these screws was bilateral as follows: frontal lobe, parietal lobe, and cerebellum. These EEG wire electrodes were routed into an EEG pedestal (Plastics One, Roanoke, VA, USA) that was secured to the skull with dental acrylic. EEG recordings were performed in awake animals with the EEG pedestal coupled to an EEG preamplifier and amplifier (Pinnacle Technologies, Lawrence, KS, USA). The raw signal was routed to a PowerLab analog-to-digital converted and data were recorded with LabChart software (AD Instruments, Colorado Springs, CO, USA) with a 60 Hz low pass filter. After each experimental session, rats were returned to their home cage and given at least 48 h in between sessions. We used a modified Racine scale for seizure severity described as follows: 0.5 = jaw clonus, 1 = myoclonic jerks, 2 = single-arm forelimb clonus, 3 = bilateral forelimb clonus and facial clonus, 4 = bilateral forelimb and facial clonus with rearing, 5 = loss of balance after rearing with forelimb and facial clonus (Piredda and Gale, 1985; Cassidy and Gale, 1998).

### Audiogenic Seizure Testing

GEPR-3 rats were first tested for response to AGSs by challenging them with 105–110 dB pure tones (Med Associates, St. Albans, VT, USA) for 60 s. In the cases of no seizure activity, the animals were given a 120 dB mixed sounds produced by an electrical bell. Following the establishment of baseline AGS score, the animals were given 60 min to recover and then tested again under a given experimental condition. The animals were then tested a final time for AGSs after an additional 20 min. Only GEPR-3s that exhibited AGSs with a score of 2 or greater were used in the pharmacological experiments. AGS seizure severity was classified into four stages as follows: 0 = no seizures, 1 = one episode of wild running seizures (WRS), 2 = two or more episodes of WRS, 3 = one episode of WRS followed by tonic-clonic seizures. Behavioral outcomes assessed included maximum seizure severity score reached, latency to seizure onset, and duration of the seizure. Seizure severity was scored by two observers in real-time and recorded with a video monitoring system (Med Associates, St. Albans, VT, USA).

### Statistics and Data Analysis

Statistical analyses were performed in GraphPad Prism version 6 (GraphPad Software, Inc., La Jolla, CA, USA) and SPSS version 25 (IBM). Non-parametric data and data that failed tests of normality (e.g., seizure severity, seizure frequency, and seizure count) were analyzed using the Wilcoxon Matched Pairs test for paired data or Friedman’s test with Dunn’s multiple comparison test.

## Results

**Figure 1:**
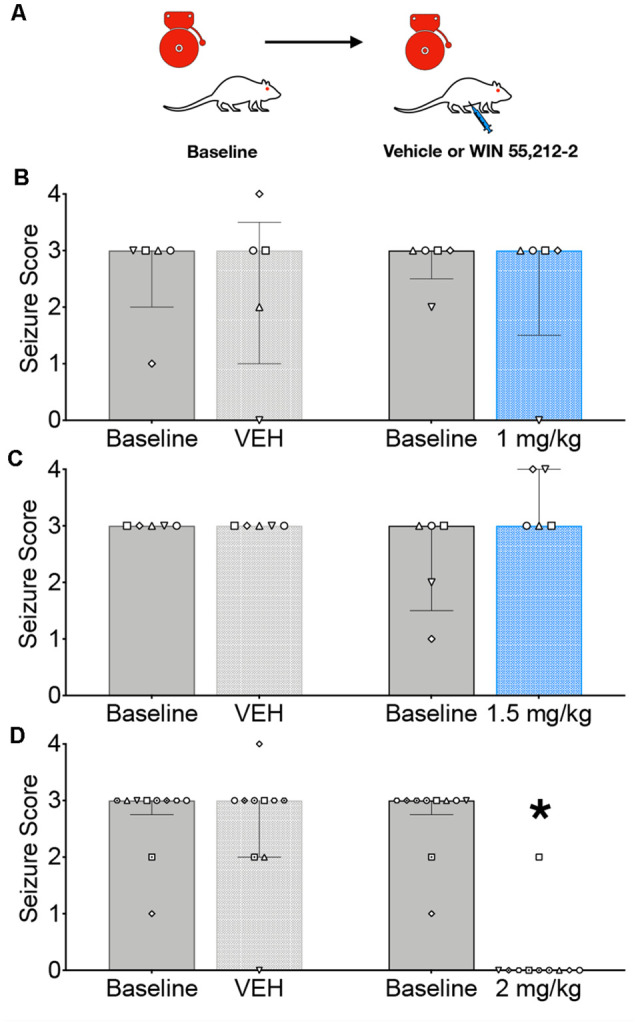
Systemic administration of the CB1/2 receptor agonist, WIN 55,212-2 (WIN) attenuates audiogenic seizures in genetically epilepsy-prone rats (GEPRs). **(A)** Schematic of the testing scheme; animals were tested with a baseline test session before a vehicle-treated session. On a separate day, animals were tested on a baseline session and then with one of three doses of WIN. The testing order was counterbalanced across subjects. **(B)** Against audiogenic seizures (AGS) scores showing baseline sessions and comparing vehicle (left) and 1 mg/kg WIN (right). **(C)** AGS scores showing baseline sessions and comparing vehicle (left) and 1.5 mg/kg WIN (right). **(D)** AGS scores showing baseline sessions and comparing vehicle (left) and 2 mg/kg (right). Bars indicate median and interquartile range, symbols show individual subjects. *p < 0.05, according to Friedman test followed by Dunn’s *post hoc* test.

We first evaluated the ability of systemic administration of WIN to attenuate AGSs in GEPR-3s; the testing strategy is shown in Figure 1A. GEPR-3s were first tested for a baseline AGS susceptibility. Thirty minutes later, GEPR-3s were injected with either vehicle or WIN (1, 1.5, or 2 mg/kg) and re-tested for AGS 30 min after injection. As shown in Figure 1B, the 1 mg/kg dose of WIN did not alter AGS responses (Friedman’s test, *Q* = 0.625, *p* = 0.94). In all cases (Baseline 1, Vehicle, Baseline 2, and 1 mg/kg of WIN) the median seizure score was 3. We found a similar lack of effect following the 1.5 mg/kg dose of WIN (Figure 1C); the median seizure score for each group was 3, and thus did not differ as a function of treatment (Friedman’s test, *Q* = 5.0, *p* = 0.24). Unlike the lower doses, 2 mg/kg of WIN produced a robust suppression of seizure activity (median seizure = 0), concurrent with mild sedation. This significant reduction in seizure severity (*Q* = 24.97, *p* = 0.000016, Friedman’s test) was driven by the difference between the second baseline session and the 2 mg/kg treated post-test (*p* = 0.0021, Dunn’s multiple comparison test). The 2 mg/kg dose also differed significantly from the vehicle (*p* = 0.0073). Importantly, the two baseline sessions did not differ from one another (*p* > 0.999), nor did the vehicle treatment differ from the baseline (*p* > 0.999).

**Figure 2:**
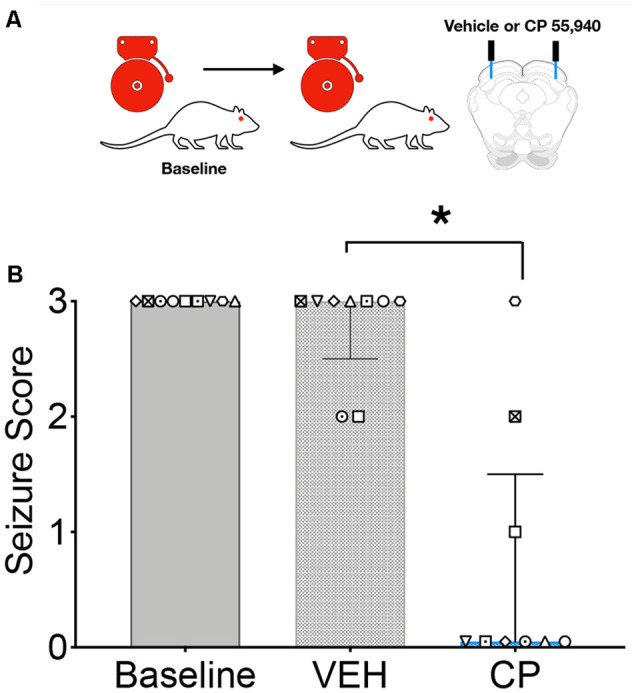
Intracollicular injection of the CB1/2 receptor agonist, CP 55940 (CP) attenuates audiogenic seizures in GEPRs. **(A)** Schematic of the testing scheme; animals were tested with a baseline test session before a vehicle-treated session and a CP treated session. A baseline is shown only once, as in this experiment all animals displayed baseline scores of three across both tests. **(B)** Microinfusion of CP significantly decreased audiogenic seizure score as compared to vehicle (VEH) infusion into the deep layers of the superior colliculus (DLSC). Bars indicate median and interquartile range, symbols show individual subjects. *p < 0.05, according to Friedman test followed by Dunn’s *post hoc* test.

We next sought to determine if focal activation of CB1 receptors in the DLSC would impair the effect of systemic drug administration; the testing scheme for these experiments is shown in Figure 2A. GEPR-3s were tested for baseline AGS susceptibility and then injected with vehicle or CP in a counterbalanced manner. During the un-injected (baseline) audiogenic susceptibility test and the vehicle injection test, the median seizure score was a 3. However, CP injection produced a significant suppression of seizure responses (median = 0; *Q* = 15.08, *p* = 0.0002; Friedman’s test, Figure 2B). Baseline and vehicle-injected conditions did not differ from one another (*p* > 0.999, Dunn’s multiple comparison test). CP injection, by contrast, significantly reduced AGS as compared both to the baseline session (*p* = 0.0065) and the vehicle-injected session (*p* = 0.029).

**Figure 3:**
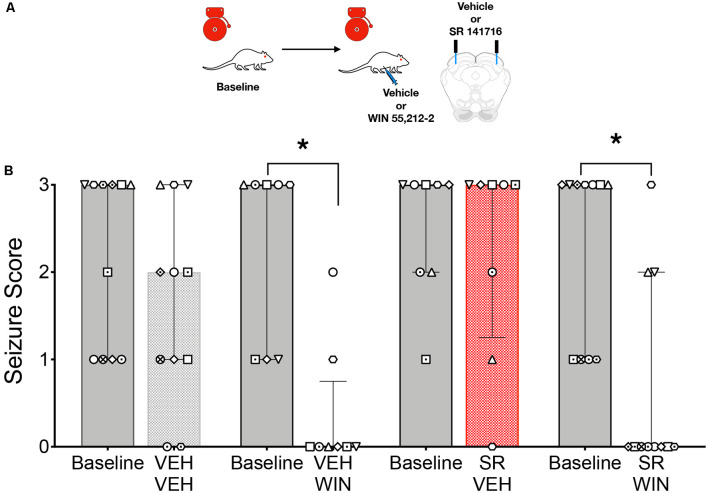
Activation of collicular CB1/2 receptors is sufficient, but not necessary to account for the anticonvulsant effect of systemic WIN 55,212-2 (WIN) in GEPRs. **(A)** Schematic of the testing scheme; animals were tested with a baseline test session before each of the four test conditions. Animals received either vehicle (VEH) or SR 141716 (SR) infusion into the DLSC followed by VEH or WIN (2 mg/kg, i.p.). **(B)** AGS score as a function of drug treatments. WIN administration significantly reduced AGS severity as compared to the pre-administration baseline. SR administration was without effect on audiogenic seizures (AGSs) but failed to reverse the anticonvulsant action of WIN. Bars show median and interquartile range, symbols show individual subjects. *p < 0.05, according to Friedman test followed by Dunn’s *post hoc* test.

Given that focal delivery of CB1/2 agonist to the DLSC resulted in a suppression of seizures that was reminiscent of that seen after systemic administration of agonist, we next sought to determine if activation of CB1/2 receptors in the DLSC was necessary for the seizure-suppressive effects of systemic drug treatment. The experimental scheme is shown in Figure 3A. GEPR-3s were tested on a baseline AGS test and then retested following systemic administration of vehicle or WIN paired with an intra-DLSC injection of vehicle or the CB1 receptor antagonist SR141716. Due to premature implant loss, not all animals received all treatments; we, therefore, analyzed these sessions independently. When the systemic vehicle was paired with the intra-DLSC vehicle, seizure responses did not differ from the baseline session (*W* = −11, *p* = 0.25, Figure 3B). Similar to what we previously observed (Figure 1), systemic administration of WIN when paired with an intra-DLSC vehicle infusion produced a significant reduction in seizure severity when compared to the baseline session (*W* = −36, *p* = 0.0078). Intra-DLSC administration of the CB antagonist SR141716, when paired with systemic vehicle injection was without effect on seizure severity as compared to the baseline session (*W* = −2, *p* = 0.75). By contrast, we observed robust seizure suppression following systemic administration of WIN, paired with intra-DLSC administration of SR141716 (*W* = −55, *p* = 0.002). Thus, blockade of CB receptors in the DLSC did not alter the anticonvulsant action of WIN.

**Figure 4:**
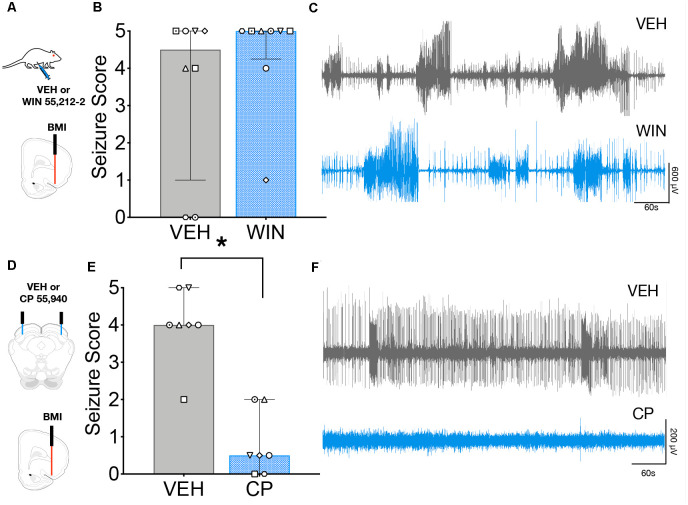
Intracollicular, but not systemic cannabinoid (CB) receptor activation suppresses seizures evoked from Area Tempestas (AT). **(A)** Seizures were evoked by injection of the GABA-A receptor antagonist, bicuculline methiodide (BMI) into the AT after systemic administration of VEH or WIN 55,212-2. **(B)** Behavioral seizure severity under the vehicle (VEH) and WIN treated conditions did not differ. **(C)** Representative electrographic seizures from the same subject under VEH and WIN treated conditions, showing multiple high amplitude events and isolated interictal spiking. **(D)** Seizures were evoked by injection of BMI into the AT following either VEH or CP infusion into the DLSC. **(E)** Seizure severity was significantly reduced by CP infusion into the DLSC. **(F)** Representative electrographic activity following treatment of the DLSC with VEH (top) and CP (bottom) from the same subject, showing that behavioral seizure suppression co-occurred with suppression of electrographic seizure activity. Bars show median and interquartile range, symbols show individual subjects. *p < 0.05.

To determine if this anticonvulsant profile held for a seizure model reliant on forebrain networks, we examined the effect of systemic (Figure 4A) and intra-DLSC (Figure 4B) administration of CB1/2 agonist against seizures evoked focally from the AT. We used a high dose of WIN (2 mg/kg) for these experiments. We found that while this dose produced mild sedation, it did not suppress behavioral (Figure 4B) or electrographic (Figure 4C) seizures. Systemic injection of the vehicle was associated with a median seizure severity of 4.5, whereas WIN administration at the dose of 2 mg/kg was associated with a median seizure score of 5 (*W* = 13, *p* = 0.41). In contrast to the lack of effect following systemic administration of WIN, intra-DLSC injection of CP was associated with robust suppression of AT-evoked seizures. Following vehicle injection into the DLSC (Figure 4D), the median AT evoked seizure response was 4 (Figure 4E). By contrast, after the intra-DLSC injection of CP, the median response was 0.5, an effect that reached the level of statistical significance (*W* = −28, *p* = 0.016). Thus, unlike the case of AGSs, systemic CB agonists did not protect against AT-evoked seizures, but intra-DLSC activation of CB receptors potently suppressed behavioral (Figure 4E) and electrographic (Figure 4F) seizures.

## Discussion

Here we report a surprising dissociation between the effects of CB agonists when administered systemically and intra-DLSC across two models of experimental seizures. While systemic administration of the CB1/2 receptor agonist WIN exerted a dose-dependent suppression of AGSs in GEPR-3s, systemic administration was without effect against seizures evoked from AT. By contrast, intra-DLSC administration of the CB1 receptor agonist CP was effective in both models. Interestingly, while activation of the CB1 receptor in the DLSC was *sufficient* to suppress seizure activity, these CB1 receptors are *not necessary*—for the anticonvulsant action of WIN.

CB receptor agonists have been widely studied for anti-seizure effects across an array of models of seizures; this has been reviewed extensively elsewhere (Wallace et al., 2001; Skaper and Di Marzo, 2012; Cristino et al., 2020). Our results are generally consistent with previous reports examining the effect of WIN on seizure susceptibility in two other AGS models. For example, a single dose of WIN failed to suppress acute AGS in Krushinsky-Molodkina rats, it delays the development of audiogenic kindling suggestive of an antiepileptogenic effect (Vinogradova and van Rijn, 2015). Furthermore, chronic treatment with CB1 antagonist SR141716A worsens AGS (Vinogradova et al., 2011). While the GEPR-3 and the Krushinsky–Molodkina rat share phenotypic similarities, we cannot exclude a contribution of strain to the differences between published reports and our present studies. Our results are likewise consistent with the findings of Citraro et al. (2016) who reported that WIN reduced AGS severity in DBA/2 mice. Similarly, activation of CB1 receptors protected against acute clonic and generalized tonic-clonic seizures in pentylenetetrazole model (Bahremand et al., 2008b) and maximal electroshock test (Wallace et al., 2001; Luszczki et al., 2011; Florek-Luszczki et al., 2014b; Florek-Luszczki et al., 2015). Thus, activation of CB1 receptors is effective in suppressing seizures that require hindbrain circuitry.

In the present study, we found that systemic administration of WIN did not alter the occurrence of AT-evoked seizure. By contrast, systemic administration of WIN reduced the incidence of spontaneous seizures in the post-status epilepticus pilocarpine model (Wallace, 2003; Di Maio et al., 2015), and reduced the seizure severity of acute pilocarpine-induced status epilepticus (Colangeli et al., 2019; Di Maio et al., 2019). Moreover, WIN protects against acute temporal-lobe like seizures in the maximal dentate activation model (Carletti et al., 2016). The AT model differs from pilocarpine in both its focal nature (vs. systemic pilocarpine) and its pharmacology (GABA antagonist vs. muscarinic agonist). It also differs in the locus of seizure origination (piriform cortex) as compared to the maximal dentate activation model (dentate gyrus). Of particular note is the dense expression of CB1 receptors on interneurons of the anterior piriform cortex (Terral et al., 2019), notably this area of dense receptor expression overlaps with the region functionally defined as AT. This raises the possibility that systemic administration of WIN may have further *disinhibited the* piriform cortex, negating anticonvulsant actions occurring at other sites in the brain.

We had hypothesized that the DLSC might be a critical site of action for CB-mediated anti-convulsant effects. This was based on: (1) the well-established role the DLSC plays in audiogenic seizures, and in particular, in the wild-running phase which is typical of the GEPR-3 strain (Merrill et al., 2005); (2) volumetric changes in the SC in GEPR-3 rats (Lee et al., 2018); (3) the interconnections between the substantia nigra pars reticulata and the DLSC and the density of CB1 receptors in the SNpr (Herkenham et al., 1991; Matsuda et al., 1992; Glass et al., 1997; Tsou et al., 1998); (4) the presence of CB1 immunopositive axons in the DLSC (Tsou et al., 1998; Sañudo-Peña et al., 2000); (5) the anticonvulsant effects of activation of the DLSC (Soper et al., 2016); and (6) the anticonvulsant effects of disinhibition of the SC through inhibition of nigrotectal projections (Wicker et al., 2019). Consistent with our hypothesis, we found that the focal application of CP to the DLSC suppressed both AGSs and AT-evoked seizures. These findings are, however, generally consistent with a *disinhibitory* action of CP in the DLSC. While we found that focal microinjection of CB1/2 agonist into the DLSC attenuated both AGSs and AT-evoked seizures, we found that the focal blockade of CB1 receptors in the DLSC was without effect on the anticonvulsant action of systemic agonist, suggesting an extra-DLSC site of the action of the agonist. A potential site of interest is the inferior colliculus that is critical in AGS initiation (see Faingold et al., 2014 for a review of audiogenic seizure networks), however, while there is an expression of CB1 receptors in the IC, the expression is less than that observed in the SC (Herkenham et al., 1991).

While the CB1 receptor is the predominant CB receptor in the brain, the CB2 receptor is also expressed in the brainstem (Van Sickle et al., 2005), cerebellum (Ashton et al., 2006; Suárez et al., 2008), and other brain regions (Gong et al., 2006). While selective CB1 receptor agonists and mixed CB1/CB2 receptor agonists have well-documented anticonvulsant effects, selective CB2 agonists are both less studied and less robust (for review see: Rosenberg et al., 2017). While WIN and CP are mixed CB1/2 agonists, the profile of anticonvulsant effects seen with WIN is similar to those of the highly selective CB1 agonist, ACEA (Rosenberg et al., 2017), suggesting that anticonvulsant effects are CB1, rather than CB2 mediated. Moreover, CB2 receptor expression in the SC (Gong et al., 2006; Duff et al., 2013) has been predominantly detected in the visual (superficial) as compared to the deep and intermediate layers, which are the sites associated with anticonvulsant action (Gale et al., 1993; Redgrave et al., 1993). Thus, it is unlikely that the anticonvulsant effects of intracollicular CP are mediated by CB2 receptors.

In sum, our data suggest that focal modulation of CB receptors within the DLSC controls both brainstems- and forebrain-evoked seizures, but also show that this site of action is not necessary for the anticonvulsant effect of systemic CB1/2 receptor agonists. While in the brainstem model, both systemic and intra-DLSC drug treatment reduced seizures, in the forebrain model, intra-DLSC drug infusion but not systemic drug treatment reduced seizures. These data add to our understanding of potential sites of action of CBs in the context of anti-seizure therapy.

## Data Availability Statement

The raw data supporting the conclusions of this article will be made available by the authors, without undue reservation.

## Ethics Statement

The animal study was reviewed and approved by Georgetown University Animal Care and Use Committee.

## Author Contributions

VS and RH contributed equally. VS, RH, PN’G, and PF designed research. VS, RH, and EW performed experiments. VS, RH, and PF analyzed data. VS, RH, and PF drafted the manuscript, which all authors edited. PN’G and PF obtained funding and supervised the study. All authors contributed to the article and approved the submitted version.

## Conflict of Interest

The authors declare that the research was conducted in the absence of any commercial or financial relationships that could be construed as a potential conflict of interest.
